# Immunogenicity and vaccine efficacy of *Actinobacillus pleuropneumoniae*-derived extracellular vesicles as a novel vaccine candidate

**DOI:** 10.1080/21505594.2025.2453818

**Published:** 2025-01-20

**Authors:** Su Hyun Park, Yun Hye Kim, Hyeon Jin Lee, Jeong Moo Han, Byoung-Joo Seo, Gyeong-Seo Park, Chonghan Kim, Young Bae Ryu, Woo Sik Kim

**Affiliations:** a Biological Resource Center, Korea Research Institute of Bioscience and Biotechnology, Jeongeup, Korea; b Functional Biomaterial Research Center, Korea Research Institute of Bioscience and Biotechnology, Jeongeup, Republic of Korea; c Department of Food and Nutrition, Chungnam National University, Daejeon, Republic of Korea; d Department of Medical Science, College of Medicine, Chungnam National University, Daejeon, Republic of Korea; e Department of Microbiology and Immunology, College of Medicine, Seoul National University, Seoul, Republic of Korea; f Institute for Data Innovation in Science, Seoul National University, Seoul, Republic of Korea; g Vaccine Lab, WOOGENE B&G Co. LTD, Seoul, Republic of Korea

**Keywords:** *Actinobacillus pleuropneumoniae*, extracellular vesicle, immunogenicity, Th1-dominant cellullar immunity, Th1-dominant humoral immunity, pre-exposure vaccine

## Abstract

*Actinobacillus pleuropneumoniae* (APP) is a significant pathogen in the swine industry, leading to substantial economic losses and highlighting the need for effective vaccines. This study evaluates the potential of APP-derived extracellular vesicles (APP-EVs) as a vaccine candidate compared to the commercial Coglapix vaccine. APP-EVs, isolated using tangential flow filtration (TFF) and cushioned ultracentrifugation, exhibited an average size of 105 nm and a zeta potential of −17.4 mV. These EVs demonstrated stability under external stressors, such as pH changes and enzymatic exposure and were found to contain 86 major metabolites. Additionally, APP-EVs induced dendritic cell (DC) maturation in a Toll-like receptor 4 (TLR4)-dependent manner without cytotoxicity. APP-EVs predominantly elicited Th1-mediated IgG responses in immunized mice without significant liver and kidney toxicity. Contrarily, unlike Coglapix, which induced stronger Th2-mediated responses and notable toxicity. In addition, APP-EVs triggered APP-specific Th1, Th17, and cytotoxic T lymphocyte (CTL) responses and promoted the activation of multifunctional T-cells. Notably, APP-EV immunization enhanced macrophage phagocytosis and improved survival rates in mice challenged with APP infection compared to those treated with Coglapix. These findings suggest that APP-EVs are promising vaccine candidates, capable of inducing potent APP-specific T-cell responses, particularly Th1, Th17, CTL, and multifunctional T-cells, thereby enhancing the protective immune response against APP infection.

## Introduction

Extracellular vesicles (EVs) secreted or released from microorganisms are non-replicative nanostructures ranging in size from 10 to 500 nm, encapsulated by a lipid bilayer membrane [1–3]. These bacteria-derived EVs (B-EVs) are categorized based on their origin as outer membrane vesicles (OMVs) from Gram-negative bacteria and cytoplasmic membrane vesicles (CMVs) from Gram-positive bacteria [4]. B-EVs play crucial roles in microbial survival and inter- and intra-species communication through their bacterial constituents, such as proteins, lipids, nucleic acids, and polysaccharides [1].

Recent research has focused on the potential of B-EVs in disease diagnosis, vaccine development, and therapeutic applications due to their functional and biochemical properties, leading to promising candidates in each field [2,5,6]. The potential of B-EVs in these medical applications is largely attributed to their bacterial components [6–8]. For instance, mass spectrometry analysis of EVs isolated from the serum of patients infected with *Mycobacterium tuberculosis* (Mtb) revealed the presence of Mtb-specific peptides, absent in healthy individuals, highlighting the potential of B-EVs as diagnostic candidates for infectious diseases [9]. In addition, EVs derived from *Lactobacillus plantarum* have shown protective effects against ischaemic brain damage attributed to the microRNAs within these EVs [10]. Furthermore, B-EVs derived from pathogenic bacteria contain various antigenic components (proteins, toxins, and polysaccharides) specific to the pathogens, which can have high antigenicity [1]. This property can be a driving force for inducing a pathogen-specific immune response when using B-EVs as a multi-antigenic vaccine in the host, making them a promising candidate for vaccine development [1,11]. Therefore, effective strategies to utilize B-EVs for diagnosis, treatment, and vaccines and a thorough analysis of their physiological functions may establish a new paradigm for B-EVs in medical applications.

This study aims to demonstrate that B-EVs, as vaccine candidates for pathogens harmful to industrial animals, have the potential to supplement and improve the immunological limitations and toxicological issues of existing vaccines. We selected *Actinobacillus pleuropneumoniae* (APP), a prominent pathogen in swine, to verify our hypothesis [12]. APP infection can cause porcine pleuropneumonia, a highly contagious disease that may lead to systemic infection and multiple organ failure [13]. This condition has significant economic implications due to reduced productivity and increased mortality rates [12–14]. Importantly, APP has shown resistance to recent antibiotics and has multiple serotypes, necessitating the development of new vaccine approaches [12,14,15]. In this study, we investigated the immunogenicity of APP-derived EVs (APP-EVs). We compared their vaccine effectiveness in systemic infection mouse model with that of an existing commercial vaccine, now referred to as “Coglapix,” which contains bacterins and toxoids of APP. Ultimately, our goal is to compare and analyze the clinical profiles of these vaccines to propose a new, more effective, and safer vaccine platform.

## Material and methods

### Ethical statements

All animal experiments, including immunization and infection studies, were conducted in accordance with the guidelines approved by the Institutional Animal Care and Use Committee of the Korea Research Institute of Bioscience and Biotechnology (KRIBB) and the Korea Research Institute for Veterinary Biologics (KRIVB). The approval number for the APP immunization experiment is KRIBB-AEC-24094. The approval number for the APP infection experiment is WG-IACUC-2024-002. The study adhered to the ARRIVE guidelines.

### Isolation of EVs from APP culture supernatants

APP-EVs were extracted from bacterial culture supernatants using a three-step process involving differential centrifugation, tangential flow filtration (TFF) system connected to a 500-kDa membrane filter, and ultracentrifugation with an iodixanol cushion. First, APP serotype 5 strain (JBNU-VDC Case no. 19–0100) supplied by Jeonbuk National University-Veterinary Diagnostic Center (JBNU-VDC, Jeollabuk-do, Republic of Korea) was inoculated in 1 L of tryptic soy broth (BD Bioscience, San Diego, CA, USA) supplemented with 5 mg/L nicotinamide adenine dinucleotide (Sigma-Aldrich, Louis, MO, USA) and cultured for 16 hours at 37°C with shaking at 160 rpm. Differential centrifugation steps were performed to collect the culture supernatants and remove pellets: the first centrifugation was conducted at 1800× g for 20 minutes, the second at 3500× g for 20 minutes, and the third at 10,000× g for 20 minutes. The supernatants were then filtered through a 0.2-µm filter (ThermoFisher Scientific, Waltham, MA, USA) to eliminate microbial contaminants. For further concentration, the filtered supernatants were processed using a TFF system (Pall Life Sciences, Port Washington, NY, USA), reducing the volume from 1 L to 16 mL. Cushioned ultracentrifugation was performed by mixing the concentrated supernatants (16 mL) with 1 mL of Optiprep solution (60% iodixanol; StemCell Technologies, Vancouver, BC, Canada) in a 17.5 mL ultracentrifuge tube (Beckman Coulter, Brea, CA, USA) and ultracentrifuging at 120,000 g for 60 minutes at 4°C to obtain high-purity APP-EVs and remove protein contaminants. The APP-EVs were diluted in 1× phosphate-buffered saline (PBS; pH 7.0), sterilized using a 0.2-µm filter, and the EV protein concentration was measured with a BCA protein assay kit (ThermoFisher Scientific). The DNA concentration within the EVs was measured was measured using Exosome DNA Extraction Kit (Creative Biolabs, Ipswich, MA, USA) according to the manufacturer’s protocol. The total lipid concentration within the EVs was measured using a Cellytic nuclear extraction kit (Abcam, Waltham, MA, USA) according to the manufacturer’s protocol. In APP-EV samples with a protein concentration of 50 µg, the total DNA and total lipid contents were measured to be 255 ng and 594.3 µg, respectively.

### Characterization and stability of APP-EVs

The particle size, zeta potential and size distribution of APP-EVs were analyzed using qNano-Exoid (IZON, Christchurch, New Zealand). This device can analyze based on Tunable Resistive Pulse Sensing. Nanosize and zeta potential measurements were analyzed according to the manufacturer’s protocol using NP150 nanopore (measuring 70–420 nm). APP-EVs resuspended in PBS (approximately pH 7.0) were exposed to acidic (pH 2.0) and alkaline (pH 12.1) environments and incubated at 37°C for 1 hour to verify the stability of APP-EVs under varying pH conditions. Following this incubation, the size, zeta potential, and size distribution of the APP-EVs were measured. Additionally, to assess stability under various enzymatic treatments, PBS-resuspended APP-EVs were treated with 3 μg/mL DNase (ThermoFisher Scientific), 6 μg/mL RNase (BioLabs, Ipswich, MA, USA), and 100 μg/mL Proteinase K (PK; BioLabs), incubated at 37°C for 1 hour. Finally, to obtain purified APP-EVs, ultracentrifugation was performed at 120,000 g for 60 minutes at 4°C. These purified EVs were used to evaluate their structural (particle size, zeta potential, and size distribution) and functional (for immune cells) stability in cellular contexts.

### Metabolite analysis

The metabolites of APP-EVs were analyzed using ultra-high-performance liquid chromatography with quadrupole time-of-flight mass spectrometry (UPLC-Q-TOF-MS) and gas chromatography-mass spectrometry (GC – MS), following established protocols [16]. This method allowed for a comprehensive profiling of the metabolites present in APP-EVs.

### Analysis of dendritic cells (DCs) maturation and viability

DCs were generated from the bone marrow cells of wild-type (WT), Toll-like receptor 2 knockout (TLR2^−/−^), and Toll-like receptor 4 knockout (TLR4^−/−^) mice, which were all on a C57BL/6 background and 8 weeks old. The differentiation process was carried out as previously described [16]. WT mice were obtained from Orient Bio (Seongnam, Republic of Korea), and TLR2^−/−^ and TLR4^−/−^ mice were purchased from Jackson Laboratory (Bar Harbor, ME, USA). Bone marrow cells were induced to differentiate into DCs over 8 days. The resulting DCs (1.5 × 10^6^ cells/well) were then treated with APP-EVs at 0.5 and 1 µg/mL concentrations and cultured for 18 hours in a 37°C CO_2_ incubator. The stimulated cells were then labelled with the DC-specific marker anti-CD11c-PE-Cyanin7, along with DC-surface markers (anti-CD80-APC, MHC-I-PE, MHC-II-PerCp-Cy5.5 from BD Bioscience) at 4°C for 30 minutes. The surface marker expression in non or stimulated DCs was analyzed using an Attune NxT Flow Cytometer (ThermoFisher Scientific). The culture supernatants were harvested, and the levels of TNF-α, IL-6, IL-1β, IL-12p70, and IL-10 were measured using specific ELISA kits (ThermoFisher Scientific) following the manufacturer’s instructions. Cell viability of staurosporine (STS, 20 nM)- and APP-EVs-treated DCs was measured using the EZ-Cytox Cell Viability Assay Kit (DoGen Bio, Seoul, South Korea), following the manufacturer’s instructions

### Mouse immunization

APP immunization mice (C57BL/6; WT mouse, 8-week-old, weighing approximately 18 g) were maintained at a constant temperature (24 ± 1°C) and humidity (50 ± 5%) under barrier conditions in the BL-2 biohazard animal facility at the KRIBB. Animals were fed sterilized commercial mouse chow and provided water ad libitum under standard light-controlled conditions (12-h light/dark periods). For immunization, APP-EV low dose (APP-EV_low_; 50 μg protein/mouse), APP-EV high dose (APP-EV_high_; 200 μg protein/mouse) and Coglapix (commercial vaccine; 100 μL) vaccines were given intramuscularly to the mice in two doses, separated by two-week intervals. The mice were euthanized two weeks following the final vaccination, and immunoassays were conducted.

### Analysis of APP-specific IgG titers

Serum was collected from the mice two weeks after the final immunization to assess APP-specific IgG titres. APP was cultured for 18 hours in 50 mL of tryptic soy broth containing NAD, then pelleted by centrifugation (10,000× g for 20 minutes) to prepare for the APP-specific IgG titres analysis. The pellets were resuspended in cold PBS and lysed using a bead beater (Allsheng, Hangzhou City, China) under the conditions: speed 6 m/s, time 30 s for 2 cycles. The lysate was then centrifuged (10,000× g for 20 minutes) to obtain the supernatant-containing proteins, which were quantified using a BCA kit. The APP protein (named BL-APP protein) isolated from bacterial lysates were used to coat an ELISA plate at 1 μg/mL concentration in KPL Coating Solution (Seracare, Milford, MA) and incubated at 4°C for 24 hours. The plate was blocked with 1× PBS containing 0.05% Tween detergent (PBST) at 4°C for 1 hour, followed by incubation with mouse serum (diluted 1:1000 in PBST) from each group at 37°C for 1 hour. After incubation, the plate was reacted with biotin-conjugated Goat anti-Mouse IgG2c (SouthernBiotech, Birmingham, AL, USA), Rat anti-Mouse IgG2b (BD Bioscience), Rat anti-Mouse IgG1 (BD Bioscience), and Rat anti-Mouse IgG2a (BD Bioscience) at 37°C for 1 hour. Finally, the plate was reacted with Avidin-HRP (BD Bioscience) at 37°C for 1 hour, and colour development was carried out using TMB substrate (BD Bioscience), followed by absorbance measurement at 495 nm.

### Analysis of liver and kidney toxicity factors in serums

Levels of alanine transaminase (ALT; Fujifilm global, Minato-ku, Tokyo, Japan), aspartate transaminase (AST; Fujifilm global), alkaline phosphatase (ALP; Fujifilm global), and creatinine (CREA; Fujifilm global) in serum samples (10 μL per sample) from each immunized mice were measured using a fully automatic analyzer (Dri-Chem-NX500, Fujifilm global) for biochemical testing, in accordance with the manufacturer’s guidelines.

### Analysis of APP-specific T-cell responses in immunized mice

Two weeks post-final immunization, spleens were collected from the mice for the analysis of APP-specific T-cell immunity. First, spleen cells (1.5 × 10^6^ cells/well) were stimulated with BL-APP protein (5 μg/mL) for 24 hours at 37°C in an incubator, and the supernatants were collected. The collected supernatants were used to measure APP-specific T-cell responses using cytokine-specific ELISA kits (IFN-γ, IL-5, IL-17A from ThermoFisher Scientific). Additionally, spleen cells (1.5 × 10^6^ cells/well) were stimulated with BL-APP protein (5 μg/mL) in the presence of a 1× protein transport inhibitor cocktail (ThermoFisher Scientific) for 12 hours at 37°C in an incubator. The cells were then collected and stained with T-cell-specific antibodies (anti-CD3-Alexa 700, CD4-PerCp-Cy5.5, CD8-APC-Cy7 from BD Bioscience, and anti-CD25-APC from ThermoFisher Scientific) for 30 minutes at 4°C. After staining, the cells were treated with Cytofix/Cytoperm kit reagent (BD Bioscience) for 30 minutes at 4°C. Finally, the cells were stained for 30 minutes at 4°C with intracellular cytokine-detected antibodies (BD Bioscience: anti-IL-5-APC, ThermoFisher Scientific: anti-IFN-γ-PE, IL-17A-PE-Cyanin7, Foxp3-PE, TNF-α-APC, and IL-2-PE-Cyanin7). The fluorescent-labelled cells were examined with a flow cytometer.

### Flow cytometry-based opsonophagocytic assay

Bacteria (APP; 1 × 10^7^ CFU) were suspended in 10 mL of 1× PBS and stained with CellTrace CFSE Cell Proliferation Kit reagent (5 μM, Invitrogen, San Diego, CA, USA) at 37°C for 30 minutes. After staining, the bacteria were washed three times with 1× PBS and resuspended in RPMI medium (Gibco BRL, Grand Island, NY, USA) containing 10% foetal bovine serum (Gibco BRL). Subsequently, CFSE-stained APP (CFSE-APP, 2 × 10^5^ CFU/well) was added to RAW 264.7 cells (mouse macrophage cell line; 2 × 10^5^/well) in U-bottom 96-well plates and co-incubated in a shaking incubator at 37°C. Following 1 hour of co-incubation, the cells were labelled with a macrophage-specific antibody (anti-F4/80-PE from BD Bioscience) at 4°C for 30 minutes. Finally, to prevent the detection of extracellular bacteria during flow cytometry analysis, 0.2 mg/mL Trypan Blue was added, and CFSE-APP^+^F4/80^+^ cells were examined.

### Measurement of defense effect through APP challenge inoculation

Mice (C57BL/6; WT mouse, 8-week-old, weighing approximately 18 g) were maintained in the BL-2 biohazard animal facility at the KRIVB. C57BL/6 mice received two intramuscular doses of APP-EVs and Coglapix vaccines at 2-week intervals. Two weeks following the final vaccination, the mice were intraperitoneally challenged with APP serotype 5 (5 × 10^7^ CFU/200 μL). Following infection, the survival rate of the mice was monitored and recorded daily to analyze the protective effect of the immunizations.

### Statistical analysis

Data were analyzed for statistical differences using GraphPad Prism 9.0 software (San Diego, CA, USA). Comparisons between two groups were performed using an unpaired t-test, while differences among three or more groups were evaluated using a one-way analysis of variance. The significance levels were set at * *p* < 0.05, **p < 0.01, ***p < 0.001.

## Results

### Stability of APP-EVs against various external stresses

**Figure 1.**
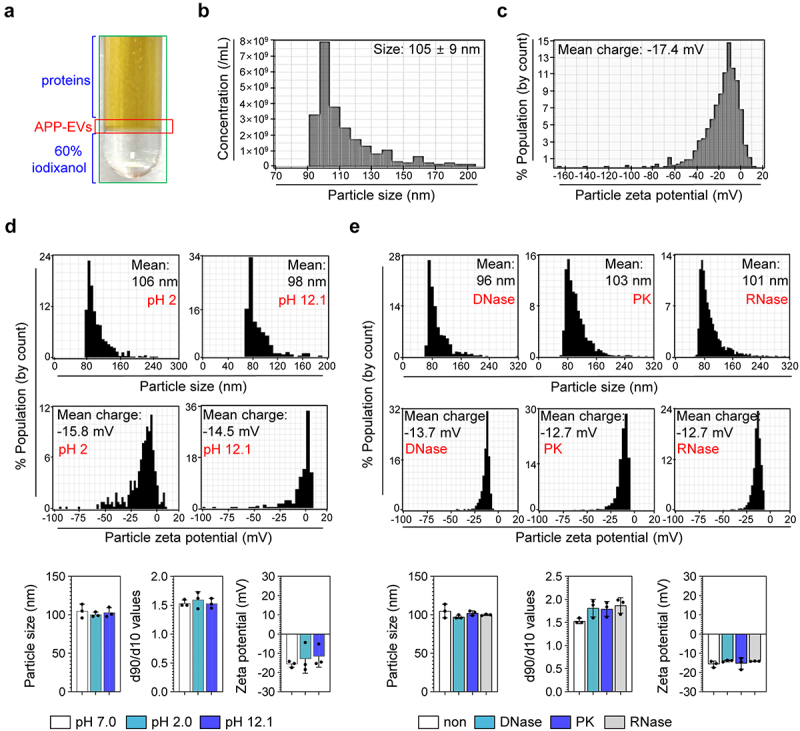
Characterization and stability of APP-EVs. (a) APP-EVs isolated via cushioned ultracentrifugation. Size (b) and zeta potential (c) of APP-EVs were analyzed using qNano-exoid. Size, zeta potential, and size distribution of APP-EVs for pH changes (d; pH 2.0 and pH 12.1) and enzyme exposures (e; DNase, RNase, PK: proteinase K) were analyzed using qNano-exoid. The qNano-exoid measurements were conducted three times, and the histograms represent typical results.

High-purity APP-EVs were obtained from the APP culture medium concentrated using the TFF system through an additional cushioned ultracentrifugation step (Figure 1a). TRPS analysis revealed that these APP-EVs have an average size of 105 nm ±9 nm (Figure 1b). The zeta potential measurement results confirmed a value of −17.4 mV (Figure 1c), within the stable range for nanoparticles (−30 mV to +30 mV). Importantly, APP-EVs did not exhibit significant differences in particle size, size distribution, or zeta potential values under alkaline (pH 12.1) and acidic (pH 2.0) conditions compared to APP-EVs in neutral conditions (pH 7.0) (Figure 1d). Additionally, when APP-EVs’ stability was assessed following exposure to DNase, RNase, and Proteinase K, the particle size, size distribution, and zeta potential values remained similar to those of APP-EVs without any stress (Figure 1e). These results suggest that APP-EVs exhibit resistance to various external stresses.

### Various metabolites constituting APP-EVs

Next, we conducted an in-depth analysis of the metabolite composition of APP-EVs using UPLC-Q-TOF-MS (Figure S1A; blue arrow, Table 1) and GC-MS (Figure S1B; red arrow, Table 2). This analysis identified 86 major metabolites within the APP-EVs in total. Specifically, UPLC-Q-TOF-MS analysis revealed 62 metabolites, including essential amino acids, peptides, nucleosides, and a diverse array of lipids, all of which play crucial roles in protein synthesis, cell signalling, metabolic regulation, and maintaining the structural integrity of the EVs. Among the amino acids identified were L-Theanine, L-Methionine, L-Phenylalanine, *N*-(1-Deoxy-1-fructosyl)phenylalanine, L-Tryptophan, Histidylleucine, Histidylcysteine, gamma-Glutamylalanine, Glutaminylvaline, L-Prolyl-L-leucine, Prolyl-gamma-glutamate, Isoleucylmethionine, and L-Isoleucyl-L-valine. The APP-EVs also contained several nucleosides, including 2’-O-Methylcytidine, Adenosine, and Guanine. The lipid composition of APP-EVs was notably diverse, comprising various phosphatidic acids (PA), phosphatidylglycerols (PG), lysophosphatidylglycerol (LysoPG), phosphatidylethanolamines (PE), lysophosphatidylethanolamines (LysoPE), lysophosphatidylcholines (LysoPC), ceramides (CerP), and N-Myristoylarginine. Key lipid molecules identified include LysoPE(20:1(11Z)), CerP(d18:1(4E)/20:0), PA(13:0/18:2(9Z,12Z)), PG(17:0/22:4(7Z,10Z,13Z,16Z)), PS(14:0/12:0), and PE(20:2(11Z,14Z)/15:0). In addition to these, we identified several fatty acids and their derivatives, including Stearoylglycine, Octadecendioic acid, Pentacosanoic acid, 13-Docosenamide, 17-Hydroxylinolenic acid, N-benzyl-9-oxo-12Z-octadecenamide, 13-Oxo-9,11-octadecadienoic acid, N-benzyl-9-oxo-10E,12E-octadecadienamide, Tetradecamide, Dodecanamide, 1-Deoxytetradecasphinganine, 1-Deoxy-11E-tetradecasphingenine, Phytosphingosine, and Adrenoyl ethanolamide. Complex lipids such as LysoPG(18:4(6Z,9Z,12Z,15Z)), CerP(d18:1(4E)/26:0), LysoPE(22:4(7Z,10Z,13Z,16Z)), LysoPG(18:0), PI(20:3(8Z,11Z,14Z)/18:0), CDP-DG(20:4(5Z,8Z,11Z,14Z)/16:0), LysoPE(22:1(13Z)), and 1-(2-methoxy-16Z-tricosenyl)-sn-glycero-3-phosphoserine were also present, highlighting the biochemical diversity and complexity of the APP-EV lipidome. Additionally, APP-EVs were found to be enriched with bioactive compounds such as Ferulic acid, a known antioxidant with immune activation properties [17]. Further, GC-MS analysis provided additional insights into the biochemical composition of APP-EVs, revealing a total of 24 metabolites. These included amino acids such as valine, alanine, leucine, isoleucine, threonine, methionine, and glutamic acid; organic acids like lactic acid, glycolic acid, succinic acid, citric acid, and oxoproline; and other significant compounds such as phosphoric acid, glucose, palmitic acid, stearic acid, myo-inositol, and lactose. This comprehensive metabolite profiling of APP-EVs underscores their complex biochemical composition, which may be critical for their functional roles in vaccine development and immune modulation.

**Table 1.** Metabolite composition of APP-EVs via UPLC-Q-TOF-ms analysis.

No.	RT (min)	Identification	Exact mass (m/z)	Fragment ions (m/z)
1	0.70	L-Theanine	175.11	84, 70
2	0.77	2’-O-Methylcytidine	258.14	129, 84, 70
3	0.88	Guanine	152.05	136, 119, 110, 84
4	1.04	L-Methionine	150.05	84, 72
5	1.12	Glutaminylvaline Histidylcysteine	246.14 259.09	129, 86
6	1.26			110
7	1.33	Histidylleucine	269.15	110
8	1.43	gamma-Glutamylalanine	219.13	130, 84, 72
9	1.46	L-prolyl-L-leucine Isoleucylmethionine	229.11 263.19	84, 72
10	1.61			132, 86
11	2.06	Adenosine Ferulic acid	268.10	136
12	2.38		195.11	167, 125
13	2.59	L-Phenylalanine	166.08	120, 103, 77
14	2.71	*N*-(1-Deoxy-1-fructosyl)phenylalanine	328.13	310, 292, 264, 120, 102
15	2.87	L-Isoleucyl-L-valine	231.17	86, 70
16	2.95	Prolyl-gamma-glutamate	244.13	147, 86
17	3.03	L-Tryptophan	205.09	146, 143, 118, 115, 91
18	3.17	LysoPE(20:1(11Z))	508.25	491
19	3.27	CerP(d18:1(4E)/20:0)	674.35	504
20	3.39	PA(13:0/18:2(9Z,12Z))	631.32	261, 197
21	3.50	N-Myristoylarginine	385.24	127
22	3.63	PG(17:0/22:4(7Z,10Z,13Z,16Z))	813.44	229
23	3.66	PS(14:0/12:0)	652.40	169
24	3.74	Stearoylglycine	342.23	183, 86, 70
25	3.83	PA(18:2(9Z,12Z)/15:0)	659.34	245
26	3.90	PG(17:2(9Z,12Z)/18:3(6Z,9Z,12Z))	755.39	491, 229
27	4.03	PE(20:2(11Z,14Z)/15:0)	730.36	488
28	4.13	LysoPG(18:4(6Z,9Z,12Z,15Z))	505.30	229
29	4.21	PA(20:3(8Z,11Z,14Z)/20:2(11Z,14Z))	751.37	263
30	4.27	CerP(d18:1(4E)/26:0)	758.41	588
31	4.37	LysoPE(22:4(7Z,10Z,13Z,16Z))	530.30	155, 127
32	4.44	LysoPG(18:0)	513.29	245, 229
33	4.66	PI(20:3(8Z,11Z,14Z)/18:0)	889.49	627, 583
34	4.89	CDP-DG(20:4(5Z,8Z,11Z,14Z)/16:0)	1002.57	229
35	5.05	PA(20:3(8Z,11Z,14Z)/20:4(5Z,8Z,11Z,14Z))	747.37	623, 245
36	5.37	LysoPG(18:1(9Z))	511.25	283, 255, 229
37	6.08	17-Hydroxylinolenic acid	295.20	229, 215
38	6.21	Tetradecamide	228.20	210, 109, 95, 81
39	6.54	N-benzyl-9-oxo-12Z-octadecenamide	386.25	244, 229, 177, 121
40	6.66	1-Deoxytetradecasphinganine	230.24	123, 95, 81, 70
41	6.92	Adrenoyl ethanolamide	376.26	293, 209
42	7.03	N-benzyl-9-oxo-10E,12E-octadecadienamide	384.25	109
43	7.07	Phytosphingosine	318.29	137, 120, 95, 81, 69
44	7.23	13-Oxo-9,11-octadecadienoic acid	295.20	229, 109, 91, 81
45	7.38	LysoPE(14:0)	426.26	285, 196, 95, 81
46	7.56			285, 155, 95
47	7.63	LysoPE(16:1(9Z))	452.27	311
48	7.81			311, 95
49	7.94	Dodecanamide	200.20	109, 81, 69
50	8.04	Octadecendioic acid	313.26	283, 215, 69
51	8.32	LysoPE(16:0)	454.29	313, 95
52	8.52			313, 282, 155
53	8.69	LysoPE(18:1(9Z))	480.30	462, 339, 155, 95, 81
54	8.73	Pentacosanoic acid	383.20	185
55	8.81	1-Deoxy-11E-tetradecasphingenine	228.22	177, 95
56	9.29	LysoPA(17:0)	425.21	309, 199, 185, 111, 95
57	9.49	LysoPE(18:0)	482.32	464, 341, 155, 95
58	10.01	LysoPC(14:1(9Z))	466.53	283
59	10.22	LysoPC(16:1(9Z))	494.57	309
60	10.49	LysoPC(18:1(9Z))	522.60	283
61	11.34	13-Docosenamide	338.34	97
62	11.96	PE(18:3(9Z,12Z,15Z)/14:1(9Z))	684.20	283
63	12.65	LysoPE(22:1(13Z))	536.20	309
64	13.70	1-(2-methoxy-16Z-tricosenyl)-sn-glycero-3-phosphoserine	610.32	221, 133, 73
65	13.96	PE(16:0/14:1(9Z))	662.47	521, 283

**Table 2.** Metabolite composition of APP-EVs via GC/MS analysis.

No.	Identification	RT (min)	RI
1	lactic acid	7.00	1054
2	glycolic acid	7.37	1071
3	valine, TMS	7.66	1085
4	alanine	7.95	1099
5	leucine, TMS	9.16	1154
6	isoleucine, TMS	9.62	1175
7	norvaline	10.42	1215
8	phosphoric acid	11.64	1292
9	norleucine	12.08	1312
10	succinic acid	12.55	1330
11	threonine	13.96	1385
12	methionine	14.51	1410
13	oxoproline	16.58	1527
14	pyroglutamic acid	16.81	1538
15	phenylalanine, TMS	17.11	1552
16	glutamic acid	18.37	1616
17	phenylalanine, 2TMS	18.49	1623
18	citric acid	21.65	1812
19	glucose	22.81	1882
20	palmitic acid	24.90	2046
21	myo-inositol	25.36	2083
22	typtophan	26.70	2202
23	stearic acid	27.13	2244
24	lactose	31.57	2744

### APP-EVs can induce TLR4-dependent DC maturation

**Figure 2.**
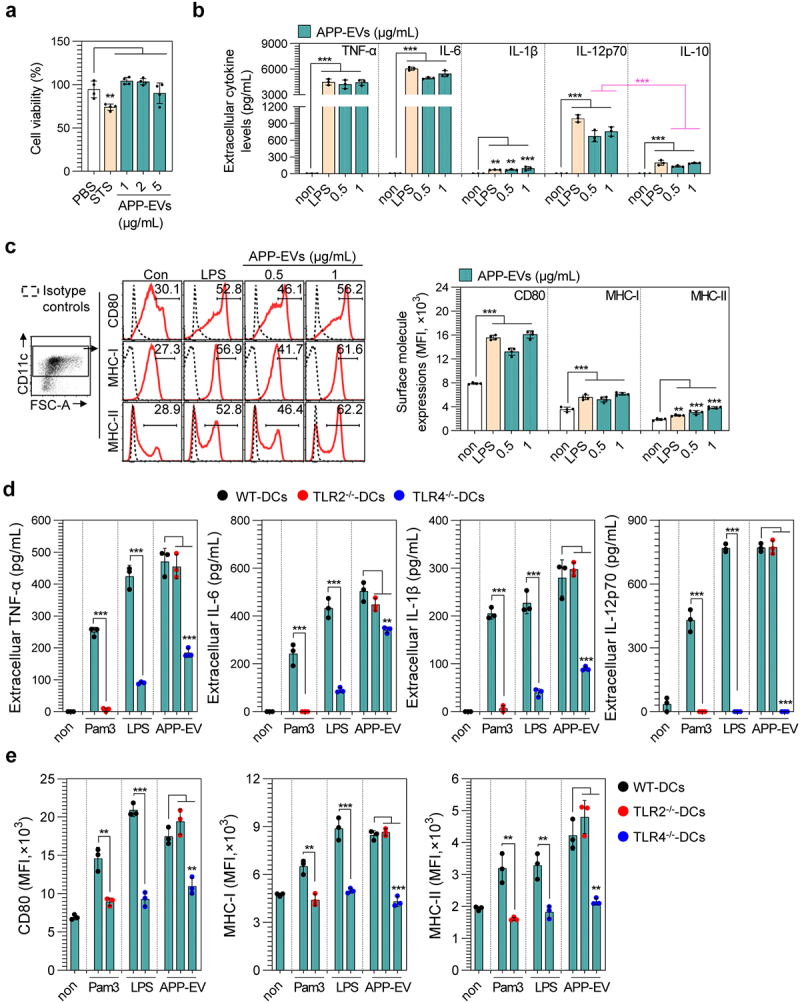
Change in cell viability, cytokine production, and surface molecule expression of DCs induced by APP-EV treatment. (a, b, c) Experiments were performed on DCs differentiated from WT C57BL/6 mice. (a) Cell viability was measured using the EZ-Cytox cell viability assay kit after treating DCs (mean ± SD; *n* = 4 samples) with staurosporine (STS, 20 nM) and APP-EVs (1, 2, and 5 μg/mL) for 18 hours. Bar graphs show mean ± SD (*n* = 4 samples). (B, C) DCs (mean ± SD; *n* = 3 samples) were treated with APP-EVs and lipopolysaccharide (LPS; 100 ng/mL) for 18 hours. (b) Cytokine levels (TNF-α, IL-6, IL-1β, IL-12p70, and IL-10) in the culture supernatants were measured using specific ELISA kits. (c) Surface molecule expression levels (anti-CD11c, -CD80, -MHC-I, and -MHC-II antibodies) were analyzed by flow cytometry, showing percentages and mean fluorescence intensity (MFI). Histograms represent typical results from three independent experiments. Statistical analysis of significant differences was performed using one-way ANOVA followed by Dunnett’s multiple comparison test for comparisons with the non-treated cell group. Statistical significance was denoted as **p < 0.01 and ***p < 0.001. (d, e) Experiments were conducted on DCs (mean ± SD; *n* = 3 samples) differentiated from WT, TLR2^−/−^, and TLR4^−/−^ mice (WT-DCs, TLR2^−/−^-DCs, TLR4^−/−^-DCs, respectively) treated with APP-EVs (1 μg/mL) for 18 hours. (d) Cytokine production levels (TNF-α, IL-6, IL-1β, and IL-12p70) in the culture supernatants were measured. (e) Surface molecule expression levels were analyzed by flow cytometry. All data represent results from two independent experiments, with representative results shown. Statistical analysis of differences between two groups was conducted using an unpaired Student’s t-test, while differences among more than two groups were evaluated using one-way ANOVA followed by Dunnett’s multiple comparison test. Statistical significance was indicated as **p < 0.01 and ***p < 0.001.

We first assessed cell viability after treating DCs with various concentrations of APP-EVs (1, 2, 5 μg/mL) for 18 hours to determine the APP-EVs’ immunological characteristics, confirming that APP-EVs were not cytotoxic up to a concentration of 5 μg/mL (Figure 2a). STS was used as a cell death inducer. The immunostimulatory activity of APP-EVs on DCs was then evaluated at non-cytotoxic concentrations (0.5 and 1 μg/mL). When DCs were stimulated with APP-EVs for 18 hours, various pro-inflammatory cytokines (TNF-α, IL-1β, IL-6), Th1 (IL-12p70)- and Th2 (IL-10)-polarizing cytokines were induced (Figure 2b). Importantly, APP-EVs induced a higher production of IL-12p70 compared to IL-10. Additionally, changes in the surface molecules of DCs were assessed, showing that APP-EVs increased the expression of CD80, MHC-I, and MHC-II in a concentration-dependent manner (Figure 2c). To assess the functional stability of APP-EVs under enzymatic stress (DNase, RNase, and Proteinase K) and pH changes, APP-EVs exposed to external stresses were applied to DCs. The results showed that APP-EVs exposed to stressors induced pro-inflammatory cytokine production and surface molecule expression similar to that of non-stressed APP-EVs (Figure S2). These findings indicate that APP-EVs maintain their immunostimulatory effects even under various external stress conditions, demonstrating their functional stability. Next, we used DCs differentiated from WT, TLR2^−/−^, and TLR4^−/−^ mice to understand further the mechanism behind DC maturation induced by APP-EVs. Thus, DCs differentiated from WT, TLR2^−/−^, and TLR4^−/−^ mice were stimulated with lipopeptide Pam3CSK4 (Pam3; 100 ng/mL), lipopolysaccharide (LPS, 100 ng/mL) and APP-EVs (1 μg/mL) for 18 hours, and then cytokine (Figure 2d) and surface levels (Figure 2a) were analyzed in cells and culture supernatants, respectively. The results showed a significant decrease in the expression of pro-inflammatory cytokines (TNF-α, IL-1β, IL-6, IL-12p70) and surface molecules (CD80, MHC-I, and MHC-II) in DCs differentiated from TLR4^−/−^ mice when treated with APP-EVs. However, DCs induced to differentiate in TLR2^−/−^ mice did not exhibit this reduction effect. Our findings suggest that APP-EVs have immunogenicity that can induce a cellular immune response (DC maturation) in a TLR4-dependent manner.

### APP-EV immunization induces app-specific Th1-mediated IgG responses without causing toxicity

**Figure 3.**
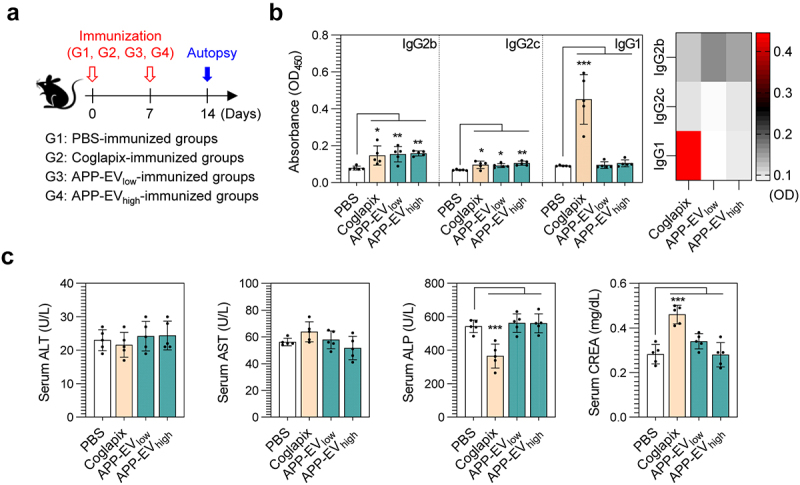
Analysis in app-specific IgG responses and toxic indicators in APP-EVs-immunized mice. (a) Immunization scheme for mice (mean ± SD; *n* = 5 mice) with Coglapix, APP-EV low dose (APP-EV_low_; 50 μg/mouse), and APP-EV high dose (APP-EV_high_; 200 μg/mouse). (b) Two weeks after the final immunization, BL-APP protein-specific IgG2b, IgG2c, and IgG1 responses in the serum of immunized mice were measured as described in the *materials and methods* section. (c) ALT, AST, ALP, and CREA levels in the serum of immunized mice were measured using a biochemical analyzer. All data represent results from two independent experiments, with representative results shown. Statistical analysis of significant differences was performed using one-way ANOVA followed by Dunnett’s multiple comparison test for comparisons with the pbs-immunized group. Statistical significance was denoted as *p < 0.05, **p < 0.01, ***p < 0.001.

APP-EVs were anticipated to exhibit superior vaccine efficacy due to their ability to activate cellular immunity. We validated this hypothesis by comparing APP-EVs’ immunogenicity with an existing commercial vaccine (Coglapix) by immunizing mice with both vaccines (Figure 3a). First, we analyzed APP-specific antibody responses in serum isolated from immunized mice. The Coglapix-immunized group induced Th1- and Th2-mediated IgG responses, specifically IgG2b, IgG2c, and IgG1. In contrast, the APP-EVs-immunized groups (APP-EV_low_ and APP-EV_high_) predominantly induced Th1-mediated IgG responses, with increased levels of IgG2b and IgG2c (Bar graphs in Figure 3b). Interestingly, the Coglapix-immunized group was confirmed to induce Th2-mediated antibody response rather than Th1-mediated antibody responses. In contrast, the APP-EVs-immunized groups (APP-EV_low_ and APP-EV_high_) were confirmed to induce Th1-dominant antibody responses (Heat map in Figure 3b). Furthermore, to evaluate the toxicological safety of the APP-EVs and Coglapix vaccines, we measured liver and kidney toxicity factors (ALT, AST, ALP, and CREA) in serum isolated from each immunized mouse two weeks after the final immunization. The values were analyzed using biochemical analysis equipment (Figure 3c). The results indicated that the APP-EV-immunized groups showed no significant changes in serum ALT, AST, ALP, and CREA levels compared to the PBS-injected groups. In contrast, the Coglapix-immunized groups showed decreased ALP levels and increased CREA levels. These findings indicate that the APP-EV vaccine can induce Th1-dominant APP-specific antibodies without causing toxicity, whereas Coglapix induces Th2-dominant APP-specific antibodies with changes in toxicity factors.

### APP-EVs immunization enhances APP-specific Th1, Th17, and CTL responses

**Figure 4.**
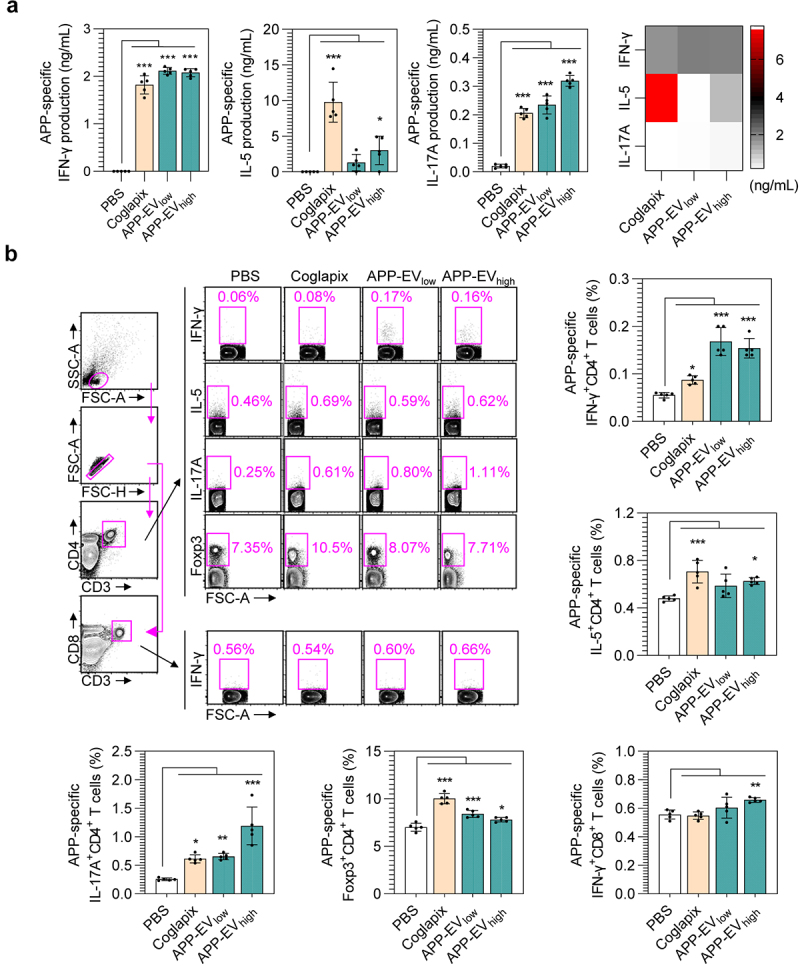
Analysis in APP-specific CD4^+^ and CD8^+^ T-cell responses in APP-EVs-immunized mice. (a) Two weeks after the final immunization, splenocytes from each mouse (mean ± SD; *n* = 5 mice) were stimulated with BL-APP protein (5 μg/mL) for 24 hours. ifn-γ, IL-5, and IL-17A levels in the culture supernatants were analyzed using cytokine-specific ELISA kits. (b) Splenocytes from each mouse were stimulated with BL-APP protein (5 μg/mL) and transport inhibitors for 12 hours. After stimulation, cells were stained with T-cell surface-specific antibodies and intracellular cytokine staining antibodies, followed by flow cytometry analysis to measure BL-APP protein-specific IFN-γ^+^CD3^+^CD4^+^ T-cells (Th1 cells), IL-5^+^CD3^+^CD4^+^ T-cells (Th2 cells), IL-17A^+^CD3^+^CD4^+^ T-cells (Th17 cells), Foxp3^+^CD3^+^CD4^+^ T-cells (regulatory T-cells), IFN-γ^+^CD3^+^CD8^+^ T-cells (CTL). All data represent results from two independent experiments, with representative results shown. Statistical analysis of significant differences was performed using one-way ANOVA followed by Dunnett’s multiple comparison test for comparisons with the pbs-immunized group. Statistical significance was denoted as *p < 0.05, **p < 0.01, ***p < 0.001.

Next, we analyzed the APP-specific T-cell responses mediated by the APP-EV vaccine. Spleen cells from immunized mice were stimulated with BL-APP protein, and extracellular cytokine levels were measured using ELISA. The APP-EVs-immunized groups (APP-EV_low_ and APP-EV_high_) showed a predominant increase in the Th1 cytokine IFN-γ rather than the Th2 cytokine IL-5. In contrast, the Coglapix-immunized group exhibited higher production of IL-5 compared to IFN-γ. Additionally, high expression of IL-17A, a cytokine that aids in neutrophil infiltration to the infection site, was observed in both APP-EVs- and Coglapix-immunized groups. However, the APP-EV_high_-immunized group demonstrated higher production of IL-17A compared to the Coglapix-immunized group (Figure 4a). Subsequently, we analyzed the activation of Th1, Th2, Th17, regulatory T-cells, and activated CD8 T-cells (cytotoxic T lymphocytes; CTLs) using flow cytometry. The APP-EVs-immunized groups (APP-EV_low_ and APP-EV_high_) exhibited higher frequencies of APP-specific IFN-γ^+^CD3^+^CD4^+^ T-cells (Th1 cells) and APP-specific IL-17A^+^CD3^+^CD4^+^ T-cells (Th17 cells) compared to the Coglapix-immunized group. A significant increase in APP-specific IFN-γ^+^CD3^+^CD8^+^ T-cells (CTLs) was observed only in the APP-EV_high_-immunized group. In contrast to the APP-EVs-immunized groups, the Coglapix-immunized group showed higher frequencies of APP-specific IL-5^+^CD3^+^CD4^+^ T-cells (Th2 cells) and APP-specific Foxp3^+^CD3^+^CD4^+^ T-cells (regulatory T-cells) (Figure 4b). These results indicate that the APP-EVs vaccine not only induces APP-specific Th1 and CTL responses but also has the potential to enhance vaccine efficacy against APP by activating APP-specific Th17 cell responses.

### APP-EVs immunization mediates activation of app-specific multifunctional CD4^+^ and CD8^+^ T-cells

**Figure 5.**
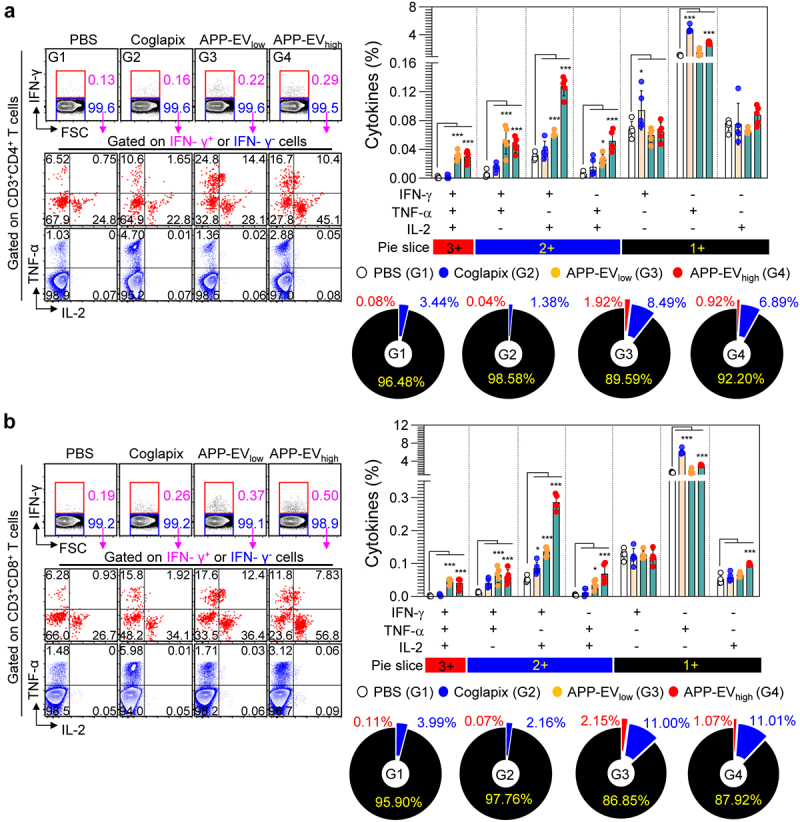
Analysis in APP-specific multifunctional CD4^+^ and CD8^+^ T-cell responses in APP-EVs-immunized mice. (a) Two weeks after the final immunization, splenocytes were stimulated with BL-APP protein (5 μg/mL) and transport inhibitors for 12 hours. After stimulation, cells were stained with T-cell surface-specific antibodies and intracellular Th1 cytokine staining antibodies. Flow cytometry gating strategy for analyzing multifunctional CD4^+^ (a; left panels) and CD8^+^ (b; left panels) T-cells producing IFN-γ, TNF-α , and IL-2 within CD3^+^CD4^+^ and CD3^+^CD8^+^ T-cell populations. Bar graphs and pie slices indicate the frequency of single-, double-, and triple-positive CD4^+^ or CD8^+^ T-cells for IFN-γ, IL-2, and TNF-α. All data represent results from two independent experiments, with representative results shown. Statistical analysis of significant differences was performed using one-way ANOVA followed by Dunnett’s multiple comparison test for comparisons with the pbs-immunized group. Statistical significance was denoted as *p < 0.05, ***p < 0.001.

APP-EV immunization has been confirmed to induce APP-specific Th1 and CTL responses. We analyzed changes in multifunctional CD4^+^ and CD8^+^ T-cells from the spleen cells of immunized mice, focusing on their potent functionality related to Th1 and CTL responses to investigate further. When spleen cells were stimulated with BL-APP protein, the APP-EVs-immunized groups (APP-EV_low_ and APP-EV_high_) exhibited higher frequencies of APP-specific triple cytokine-positive (IFN-γ^+^TNF-α^+^IL-2^+^) and double cytokine-positive (IFN-γ^+^TNF-α^+^, IFN-γ^+^IL-2^+^, TNF-α^+^IL-2^+^) multifunctional CD4^+^ T-cells compared to the PBS-injected control group (Figure 5a). Furthermore, analysis of multifunctional CD8^+^ T-cell activity revealed that the APP-EVs-immunized groups (APP-EV_low_ and APP-EV_high_) demonstrated higher frequencies of APP-specific triple cytokine-positive (IFN-γ^+^TNF-α^+^IL-2^+^) and double cytokine-positive (IFN-γ^+^TNF-α^+^, IFN-γ^+^IL-2^+^, TNF-α^+^IL-2^+^) multifunctional CD8^+^ T-cells compared to the PBS-injected groups (Figure 5b). In contrast, the Coglapix-immunized group did not show changes in the activation of multifunctional CD4^+^ and CD8^+^ T-cells (Figure 5a,b). These results suggest that APP-EVs are a potent vaccine candidate capable of inducing APP-specific multifunctional T-cell responses.

### Antibodies formed by APP-EVs immunization show strong opsonophagocytic activity against APP and increase mouse survival rate against APP infection

**Figure 6.**
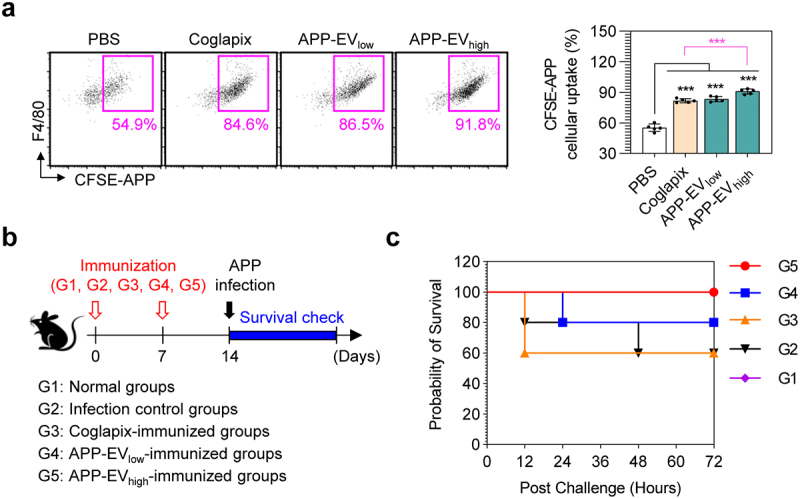
Opsonophagocytic activity and protective efficacy against APP infection induced by APP-EVs immunization. (a) Two weeks after the final immunization, cfse-labelled APP (CFSE-APP) was incubated with serum from each group of immunized mice (PBS, coglapix, APP-EV_low_, APP-EV_high_) at 37°C for 30 minutes as described in the materials and methods section. After incubation, CFSE-APP was collected and co-incubated with the raw 264.7 macrophage cell line for 1 hour. Uptake levels of CFSE-APP by raw 264.7 cells were analyzed by flow cytometry after staining raw 264.7 cells with the macrophage-specific antibody F4/80 to detect F4/80^+^CFSE-APP^+^ cells. Statistical analysis of significant differences was performed using one-way ANOVA followed by Dunnett’s multiple comparison test for comparisons with the pbs-immunized group. Statistical significance was denoted as *p < 0.05, ***p < 0.001. (b) Schematic of the experimental design for evaluating the protective efficacy of PBS, coglapix, APP-EV_low_, and APP-EV_high_ immunization against APP infection. (c) Survival data were collected timely to assess the protective efficacy of each immunization group against APP infection. All data represent results from two independent experiments, with representative results shown.

We conducted an opsonophagocytic assay using macrophages (Raw 264.7 cells) as described in the *Materials and Methods* section to determine if antibodies in the serum isolated from each immunized mouse bind to APP and facilitate effective phagocytosis by macrophages. The results showed that, compared to serum from the PBS-injected group, CFSE-APP co-incubated with serum from both the APP-EVs- and Coglapix-immunized groups led to increased phagocytosis by Raw 264.7 cells. Notably, the APP-EV_high_-immunized group exhibited higher phagocytosis rates than the Coglapix-immunized group (Figure 6a). Additionally, we demonstrated the superiority of the APP-EV vaccine by analyzing changes in mouse survival rates following APP infection two weeks after immunization with APP-EVs and Coglapix (Figure 6b). The vaccine efficacy was assessed by determining the lethal dose (LD50) through infections with varying doses of APP (1 × 10^6^ CFU, 5 × 10^7^ CFU, 1 × 10^8^ CFU, 1 × 10^9^ CFU). Following an APP infection at 5 × 10^7^ CFU, the survival rate of mice was observed to be 60% at 48 hours post-infection and remained stable up to 144 hours (data not shown). Based on these findings, we selected 5 × 10^7^ CFU as the benchmark dose for evaluating vaccine efficacy.

Interestingly, the groups immunized with low (APP-EV_low_) and high (APP-EV_high_) doses of APP-EVs exhibited improved survival rates, with 80% and 100% of the mice surviving for up to 72 hours post-infection, respectively. However, the Coglapix-immunized group failed to show any protective effect against APP infection (Figure 6c). These findings suggest that the higher protective effect of the APP-EV vaccine compared to the Coglapix vaccine against APP infection may be due to opsonophagocytic activity initiated alongside APP-EV-mediated cellular and humoral immunity.

## Discussion

This study provides a comprehensive analysis of the immunogenic and protective properties of APP-EVs as a potential vaccine candidate against APP infection, comparing its efficacy to that of the commercial vaccine Coglapix. Initially, we confirmed APP-EVs’ stability under various external stress conditions and assessed their immunogenicity using DCs, which are critical for initiating adaptive immune responses. APP-EVs demonstrated resistance to both alkaline and acidic conditions and exposure to various enzymes (DNase, RNase, and PK). Importantly, these APP-EVs were discovered to act as selective agonists of TLR4 in DCs, resulting in the high production of pro-inflammatory cytokines and elevated expression of surface molecules, thus inducing the functional maturation of DCs. Furthermore, APP-EVs, capable of inducing these immunogenic responses, were shown to elicit APP-specific Th1, Th17, CTL, and multifunctional T-cell responses in immunized mice, accompanied by Th1-dominant IgG responses (IgG2b and IgG2c). In contrast to APP-EVs, the Coglapix vaccine predominantly induced APP-specific Th2 immunity and a Th2-dominant IgG1 response. Importantly, the opsonophagocytic activity, which more effectively controls APP infection, was significantly higher in the immune system induced by APP-EV immunization compared to the Coglapix vaccine. Ultimately, APP-EVs demonstrated superior protective efficacy as a preventive vaccine against APP infection compared to Coglapix. The intraperitoneal infection model used in this study has been reported to reliably induce systemic pneumococcal infection [18]. These results underscore the potential of APP-EVs as an effective means of managing early-stage bacterial infections, particularly given APP’s capability to disseminate to multiple organs in the pig during the acute phase and its association with bacteraemia [19].

Vaccine candidates against various pathogens are based on pathogen-specific antigenicity, but most lack sufficient immunogenicity [20]. These vaccines are being developed through a formulation strategy using adjuvants to enhance the immune response [21]. For example, virus-like particles (VLPs) used as vaccine candidates against foot-and-mouth disease virus (FMDV) show a protective effect against foot-and-mouth disease, but their low immunogenicity results in inadequate T-cell and B-cell responses, which are crucial for optimal protection [22]. However, when these VLPs were formulated with adjuvants (TLR4 agonists and liposomes), better pathogen-specific T-cell and B-cell responses were generated [23]. These results highlight the importance of adjuvants in enhancing immunogenicity during vaccine development [24]. Importantly, our findings that APP-EVs induce DC maturation in a TLR4 signalling-dependent manner suggest that APP-EVs themselves can be highly immunogenic without the need for additional adjuvants. In the context of APP infection, it has been reported that APP virulence factors stimulate TLR4 signalling in the early stages of infection, leading to an inflammatory response [25]. Bacterial EVs are characterized by their ability to carry cargo from their parent cells, enabling them to modulate immune responses [26]. Thus, APP-EVs appear to induce DC maturation by activating TLR4 signalling, reflecting the unique characteristics of the APP strain. Consequently, while other TLRs may also be involved in the immunostimulatory effects of APP-EVs, our findings, combined with recent studies on the immunostimulatory capacity of APP strains, suggest that APP-EVs predominantly exert their immunogenicity through a TLR4-dependent mechanism.

Next, there is a need to pay attention to the cellular and humoral immunity induced by the APP-EV vaccine. T-cell immunity plays a central role in pathogen defence in developing a vaccine, and vaccine efficacy can vary depending on the T-cell types induced by the vaccine [27]. Effective vaccines against extracellular and Gram-negative bacteria require the induction of both strong T-cell responses, particularly Th1 and Th17, and robust B-cell responses to produce high-affinity antibodies [28,29]. Interestingly, although both the APP-EV and Coglapix vaccines can induce APP-specific humoral immunity and promote opsonophagocytic activity, there are clear differences in their protective effectiveness against APP infection. The APP-EV vaccine could promote Th1 immune-dependent antibody responses (IgG2b and IgG2c) along with APP-specific Th1 responses, ultimately activating macrophages to enhance phagocytosis and bacterial killing [30,31].

In contrast, Coglapix markedly induced Th2 responses, predominantly inducing antibody responses (IgG1) dependent on Th2 immunity, which are involved in antibody production, especially neutralizing and opsonizing antibodies. Importantly, Th1-mediated IgG2 isotype antibodies are known to be more efficient in eliminating pathogens and activating the complement system than IgG1 [32]. In addition, compared to the Coglapix vaccine, the APP-EV vaccine showed higher activity of Th17 cells, which can kill bacteria by recruiting neutrophils to the site of infection [33]. Moreover, the APP-EV vaccine has the potential to inhibit bacterial replication and spread by targeting bacteria-infected cells and inducing the activation of CTL cells [34,35]. Besides, activating multifunctional T-cells exclusive to the APP-EV vaccine suggests that APP-EVs can achieve a stronger and sustained protective effect. These antigen-specific multifunctional CD4^+^ and CD8^+^ T-cells produced by the vaccine simultaneously secrete Th1 cytokines, exhibiting superior functionality compared to Th1 and CTL cells [36,37]. Importantly, they also possess a robust and enduring memory function against pathogens. Accordingly, the induction of multifunctional T-cells has emerged as a crucial benchmark in developing vaccines against a wide range of bacterial infections [36,38,39]. These results suggest that variations in T-cell immunity induced by APP-EV and Coglapix vaccines may ultimately determine their effectiveness in protecting against APP infection.

Finally, attention must be given to toxicological safety profile verification results, which are crucial in developing treatments and vaccines. The APP-EV vaccine demonstrated no significant changes in liver and kidney function indicators (ALT, AST, ALP, CREA), whereas the Coglapix vaccine exhibited decreased ALP and increased CREA levels. Notably, low ALP can lead to nutritional deficiencies and bone formation disorders, while high CREA levels may indicate underlying health issues such as kidney infections and heart failure [40–42]. Our findings suggest that the APP-EV vaccine may offer a higher safety profile than the Coglapix vaccine.

In conclusion, our results provide a solid foundation for advancing the development of the APP-EV vaccine, potentially paving the way for future clinical trials to evaluate its efficacy and safety in preventing APP infection in swine populations. The immunogenic potency and safety demonstrated by APP-EVs suggest they could offer a more effective and safer alternative to current vaccines, addressing the significant economic impact of APP in the swine industry. However, despite these promising findings, further research is essential to develop a more successful EV-based vaccine. First, additional studies are needed to further elucidate the superior antigenicity and immunogenicity of APP-EVs by directly comparing their protective effects against various APP serotypes in infected pigs with the protective efficacy of existing commercial vaccines. Additionally, expanding the dataset on APP-EVs’ antigenicity and evaluating their effectiveness across different serotypes will provide crucial insights into the correlation between protective immunity and antigen composition. Lastly, in-depth studies on the functional and structural properties of the metabolites identified in APP-EVs are necessary. These efforts are vital for refining the development of a more effective and comprehensive EV-based vaccine, ultimately contributing to better prevention and control of APP infections in the swine industry.

## Supplementary Material

Figure S1.tif

The ARRIVE guidelines_author checklist.pdf

Figure S2.tif

## Data Availability

The data supporting the findings of this study are available at Mendeley Data (https://data.mendeley.com/datasets/ttgdhfm4gf/1 or DOI: 10.17632/ttgdhfm4gf.1).
