# Supplementary figures and images for: Immunogenicity and vaccine efficacy of Actinobacillus pleuropneumoniae-derived extracellular vesicles as a novel vaccine candidate
Source: Virulence. 2025 Jan 20;16(1):2453818. doi: 10.1080/21505594.2025.2453818 (PMC11749362; doi:10.1080/21505594.2025.2453818)

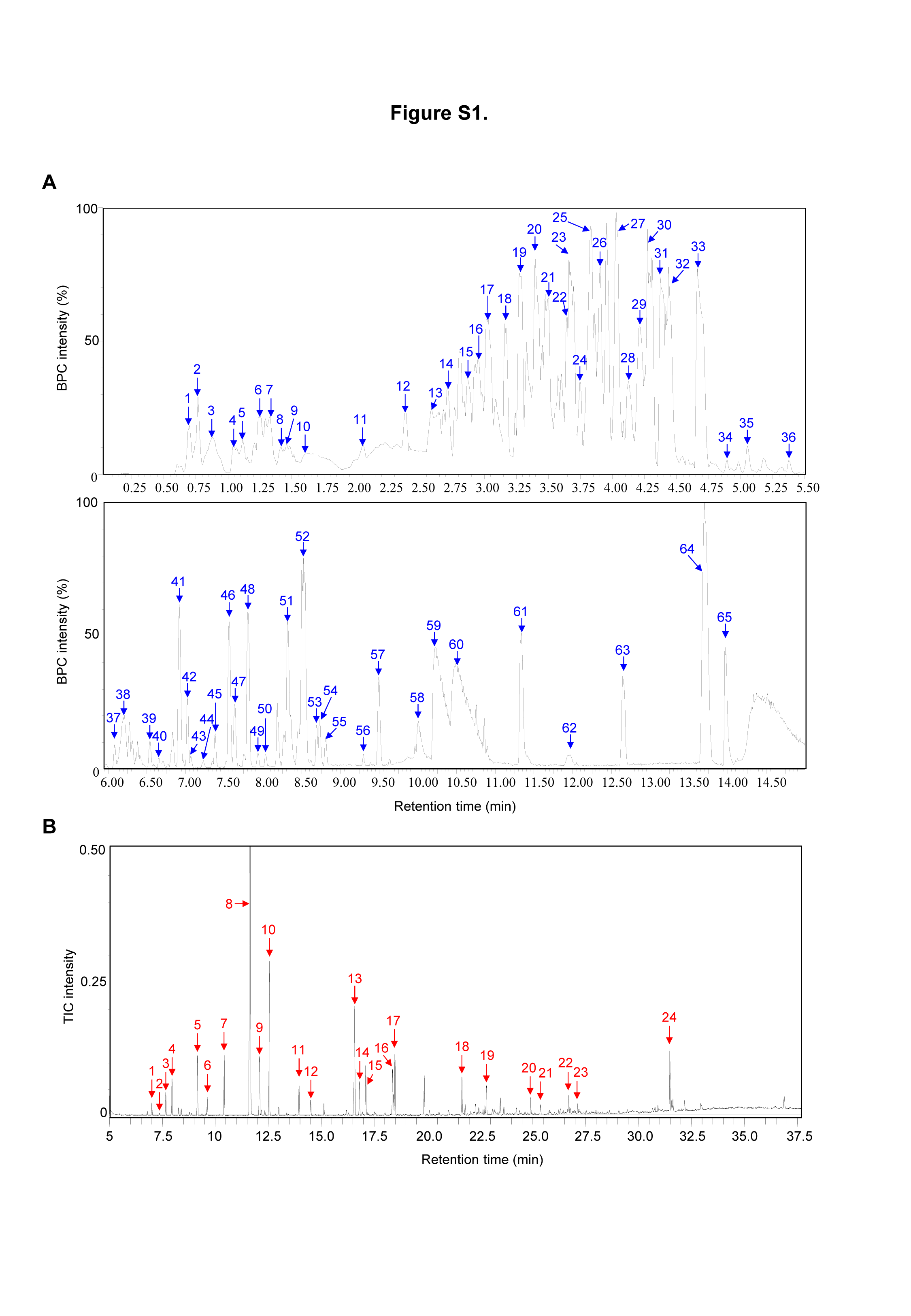

Supplement: Figure S1.tif [file KVIR_A_2453818_SM3616.tif]

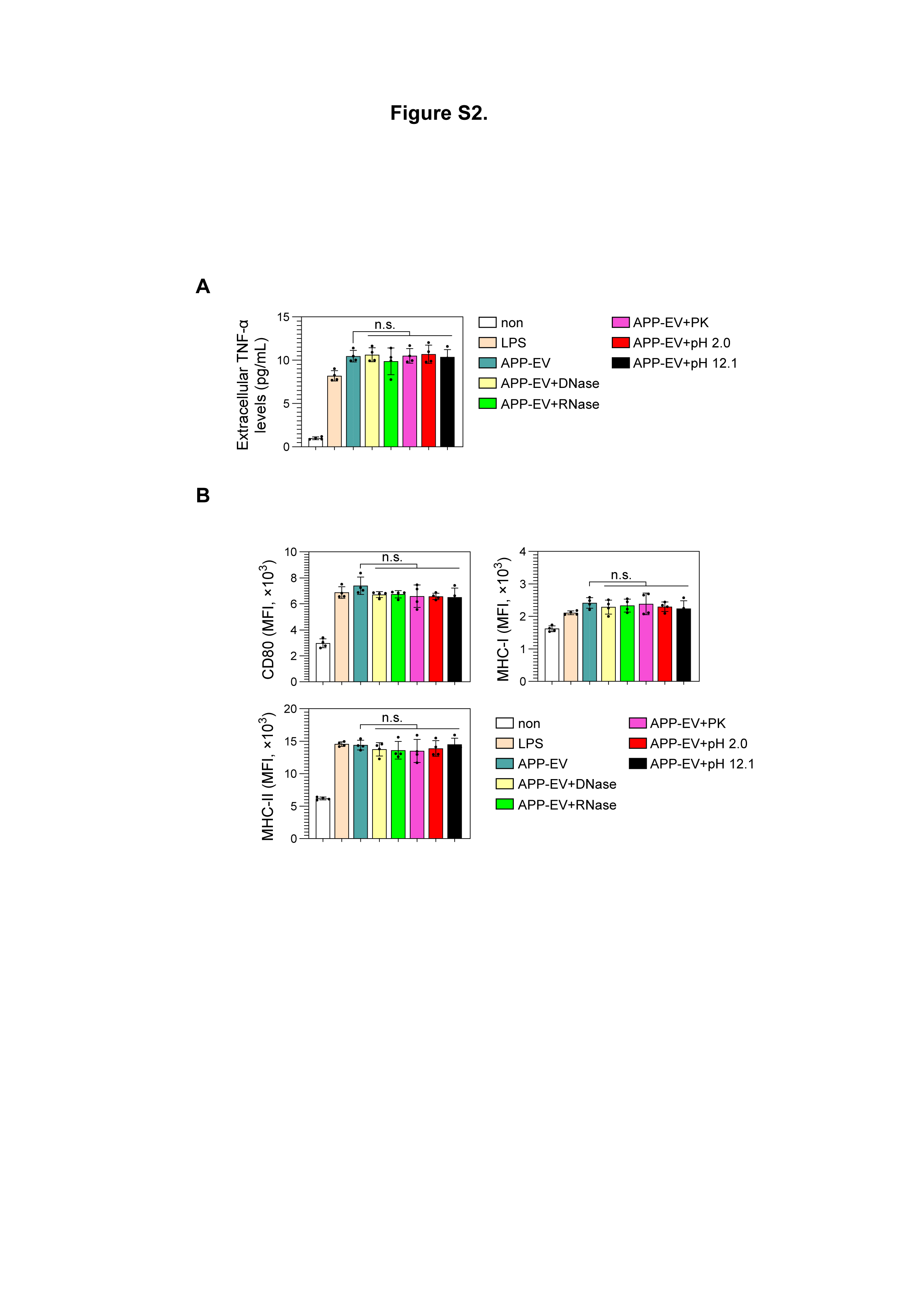

Supplement: Figure S2.tif [file KVIR_A_2453818_SM3614.tif]
